# CcpA Affects Infectivity of *Staphylococcus aureus* in a Hyperglycemic Environment

**DOI:** 10.3389/fcimb.2017.00172

**Published:** 2017-05-09

**Authors:** Markus Bischoff, Bodo Wonnenberg, Nadine Nippe, Naja J. Nyffenegger-Jann, Meike Voss, Christoph Beisswenger, Cord Sunderkötter, Virginie Molle, Quoc Thai Dinh, Frank Lammert, Robert Bals, Mathias Herrmann, Greg A. Somerville, Thomas Tschernig, Rosmarie Gaupp

**Affiliations:** ^1^Institute for Medical Microbiology and Hygiene, Saarland UniversityHomburg, Germany; ^2^Institute of Anatomy and Cell Biology, Saarland UniversityHomburg, Germany; ^3^Institute of Immunology, University of MunsterMunster, Germany; ^4^Division of Infection Biology, Department of Biomedicine, University Hospital BaselBasel, Switzerland; ^5^Department of Internal Medicine V - Pulmonology, Allergology and Critical Care Medicine, Saarland University HospitalHomburg, Germany; ^6^Department of Dermatology, University of MunsterMunster, Germany; ^7^DIMNP, CNRS, Univ MontpellierMontpellier, France; ^8^Department of Experimental Pneumology and Allergology, Saarland University HospitalHomburg, Germany; ^9^Department of Medicine II, Saarland University HospitalHomburg, Germany; ^10^School of Veterinary Medicine and Biomedical Sciences, University of Nebraska-LincolnLincoln, NE, USA

**Keywords:** *Staphylococcus aureus*, CcpA, infectivity, hyperglycemia, carbon catabolic regulation

## Abstract

Many bacteria regulate the expression of virulence factors via carbon catabolite responsive elements. In Gram-positive bacteria, the predominant mediator of carbon catabolite repression is the catabolite control protein A (CcpA). Hyperglycemia is a widespread disorder that predisposes individuals to an array of symptoms and an increased risk of infections. In hyperglycemic individuals, the bacterium *Staphylococcus aureus* causes serious, life-threatening infections. The importance of CcpA in regulating carbon catabolite repression in *S. aureus* suggests it may be important for infections in hyperglycemic individuals. To test this suggestion, hyperglycemic non-obese diabetic (NOD; blood glucose level ≥20 mM) mice were challenged with the mouse pathogenic *S. aureus* strain Newman and the isogenic *ccpA* deletion mutant (MST14), and the effects on infectivity were determined. Diabetic NOD mice challenged with the *ccpA* deletion mutant enhanced the symptoms of infection in an acute murine pneumonia model relative to the parental strain. Interestingly, when diabetic NOD mice were used in footpad or catheter infection models, infectivity of the *ccpA* mutant decreased relative to the parental strain. These differences greatly diminished when normoglycemic NOD mice (blood glucose level ≤ 10 mM) were used. These data suggest that CcpA is important for infectivity of *S. aureus* in hyperglycemic individuals.

## Introduction

Diabetes mellitus is a common endocrinopathy in both household animals and humans (Bennett, 2002; Shaw et al., 2010). A common complication in diabetes is foot ulceration, which is typically associated with limb bone and tissue infection, and incurring significant morbidity, disability, and frequent lower limb amputation (reviewed in Game, 2010; Ambrosch et al., 2011). The Gram-positive opportunistic pathogen *Staphylococcus aureus* is one of the leading causes of this disease, either as a singular microbial causative agent, and/or in synergy with other pathogenic microorganisms (Yates et al., 2009). The availability of an excellent diabetic mouse model that recapitulates the human disease has greatly facilitated research into this growing problem and should facilitate new therapeutic treatments. *S. aureus* infections in diabetic mice are associated with increased inflammation, endothelial injury, and blood coagulation (Rich and Lee, 2005; Tsao et al., 2006; Hanses et al., 2011). In addition, the killing of *S. aureus* in the diabetic host is impaired due to a diminished leukocytic respiratory burst (Rich and Lee, 2005). While these studies focused on host factors involved in the immune response, little is known about the bacterial factors that mediate *S. aureus* success in colonizing and causing infections in diabetic foot ulcerations (DFU). That being said, polysaccharide intercellular adhesion (PIA) synthesis is common in *S. aureus* isolates obtained from patients with DFU (Podbielska et al., 2010), and most *S. aureus* strains isolated from DFU are positive for the epidermal cell differentiation inhibitor EDIN (Messad et al., 2013). However, the importance of PIA and EDIN in the infectivity of *S. aureus* in DFU is unknown, and it remains to be seen whether PIA and EDIN are expressed in the diabetic environment relative to normoglycemic conditions.

PIA synthesis is encoded within the *ica* operon, and is regulated in part by the catabolite control protein A (CcpA), a glucose-responsive member of the LacI/GalR family of transcriptional regulators (Seidl et al., 2008b). CcpA also modulates transcription of exotoxins, such as α-hemolysin and toxic shock syndrome toxin 1, protein A (SpA), and capsule formation in a glucose-responsive manner (Seidl et al., 2006, 2008a, 2009). More recently, CcpA was reported to mediate proline and arginine auxotrophies under *in vitro* growth conditions (Li et al., 2010; Nuxoll et al., 2012), and to contribute to infectivity of *S. aureus* in a murine model of staphylococcal abscess formation (Li et al., 2010). CcpA regulatory activity in *S. aureus* is induced by carbon sources such as glucose, fructose, glycerol, sucrose, mannitol, maltose, and salicin (Li et al., 2010; Nuxoll et al., 2012), with as little as 4 mM of glucose being sufficient to fully activate CcpA *in vitro* (Seidl et al., 2008a). Furthermore, *S. aureus* CcpA activity is affected by the serine/threonine kinase Stk1, which inactivates CcpA via the phosphorylation of threonines 18 and 33 located in the DNA binding site (Leiba et al., 2012). The environmental stimuli that activate Stk1 to phosphorylate CcpA have yet to be identified.

The prevalence of *S. aureus* infections in hyperglycemic individuals, the preponderance of PIA positive *S. aureus* isolates from DFU patients, and the CcpA-mediated regulation of PIA, suggest that CcpA is important for infectivity of *S. aureus* in a diabetic host. To test this hypothesis, the ability of *S. aureus* strain Newman and its isogenic *ccpA* mutant to elaborate infections in three independent murine models using normo- and hyperglycemic conditions were examined.

## Material and methods

### Bacterial strains, plasmids, and culture conditions

Bacterial strains and plasmids used in this study are listed in Table 1. Bacteria were routinely grown at 37°C and 150 rpm in tryptic soy broth (TSB) purchased from Becton Dickinson (Heidelberg, Germany) with a culture to flask volume ratio of 1:10. As required, the medium was supplemented with 50 μg kanamycin per milliliter.

**Table 1:** **Strains and plasmids used in this study**.

**Strain**	**Relevant genotype and phenotype^a^**	**Reference or source**
***S. aureus***		
Newman	Mouse pathogenic laboratory strain (ATCC 25904)	Duthie, 1952
MST14	Newman Δ*ccpA::tet*(L); Tc^R^	Seidl et al., 2006
**Plasmids**		
pCN34_*ccpA*	pCN34 derivative harboring *ccpA* and its native promoter; Ap^R^ in *E. coli*/Km^R^ in *S. aureus*	Leiba et al., 2012
pCN34_*ccpA*_Ala	pCN34 with a *ccpA* derivative carrying the CcpA_T18A/T33A mutations under the control of the *ccpA* promoter; Ap^R^ in *E. coli*/Km^R^ in *S. aureus*	Leiba et al., 2012

a *Ap^R^, ampicillin resistant; Km^R^, kanamycin resistant; Tc^R^, tetracyclin resistant*.

#### Animal models

Eight week old female C57BL/6N and non-obese diabetic mice (NOD/ShiLtJ) were purchased from Charles River Laboratories (Sulzfeld, Germany) and kept under specific pathogen-free conditions according to the regulations of German and Swiss veterinary law, respectively. All animal studies were performed with the approval of the animal welfare committees Landesamt für Verbraucherschutz (Saarbrucken, Germany), Landesamt für Natur Umwelt und Verbraucherschutz (Recklinghausen, Germany), and cantonal veterinary office of Basel-Stadt (Switzerland), respectively. Female NOD mice spontaneously develop type 1 diabetes mellitus usually between 15 and 30 weeks of age (Leiter, 2001). Animals of 12 weeks of age were tested weekly for increased urinary glucose levels, and subsequently analyzed for blood glucose levels. Mice with blood glucose ≥20 mM were included as diabetic animals in the study. For all animal models, phosphate buffered saline (PBS)-washed bacterial cells obtained from exponential growth phase cultures (i.e., after 2 h of growth in TSB at 37°C and 150 rpm) were used as inocula.

The murine lung infection model was performed as described (Hartmann et al., 2014). Eight weeks old C57BL/6N mice were slightly anesthetized by intraperitoneal injection of 100 mg/kg body weight of ketamine hydrochloride (Pfizer, Berlin, Germany) and 10 mg/kg of xylazine hydrochloride (Bayer, Leverkusen, Germany), and infected intranasally with 5 × 10^7^ colony forming units (CFU) of *S. aureus*. Twenty-four hours post infection (p.i.), the animals were euthanized, the tracheae were cannulated and a broncho-alveolar lavage (BAL) was performed (three times with 1 ml of PBS). The BAL fluid was centrifuged at 300 × g for 10 min at 4°C to obtain alveolar cells, which were suspended in 1 ml of PBS. Total cell numbers in BAL were determined using a Neubauer hemocytometer. To identify the bacterial load of the lungs 24 h p.i., whole lungs were homogenized in 1 ml of PBS, and serial dilutions were plated on sheep blood agar (SBA). CFU were counted after incubation overnight at 37°C.

The footpad swelling model was carried out as described (Nippe et al., 2011). Age-matched mice were inoculated subcutaneously with 1 × 10^7^ CFU of *S. aureus* into the left hind footpad, and footpad swelling was measured daily with a micrometric caliper in reference to the uninfected footpad. Eleven days p.i., mice were killed by CO_2_ asphyxiation, and footpad tissues aseptically sampled, homogenized in PBS, and serial dilutions were plated on SBA to determine bacterial loads.

For the catheter-related biofilm infection model, mice were anesthetized with isoflurane (Baxter, Volketswil, Switzerland) and sterile catheter segments were inserted subcutaneously as described (Rupp et al., 1999). Catheters were infected with 20 μl pyrogen-free saline containing 1 × 10^4^ CFU of *S. aureus*, before the incisions were closed with wound clips. The diameter of swelling/edema was measured daily using a caliper. Ten days after infection, mice were euthanized by intraperitoneal injections of thiopenthal (500 mg/kg; Ospedalia AG, Hünenberg, Switzerland), and the catheters and surrounding tissues were aseptically removed and separated as described (Sadykov et al., 2011). Briefly, bacteria adherent to the catheters were detached by vortexing in 0.9% saline supplemented with 0.15% EDTA, followed by sonication for 2 min at 250 W. Tissue samples were homogenized in 1 ml 0.9% saline. Serial dilutions were plated on SBA to determine the bacterial loads in catheters and tissues.

The murine abscess model was performed as described by Li et al. (2010) with minor modifications. Briefly, 8 weeks old C57BL/6N mice were anesthetized by intraperitoneal injection of 100 mg/kg body weight of ketamine hydrochloride (Pfizer) and 10 mg/kg of xylazine hydrochloride (Bayer), and infected with 1 × 10^7^ CFU of *S. aureus* via retro-orbital injection. Infected mice received once a day an intraperitoneal injection of the non-steroidal anti-inflammatory agent caprofen (5 mg/kg; Pfizer) as pain relief, and 4 days p.i., mice were euthanized with pentobarbital (400 mg/kg; Merial GmbH, Hallbergmoos, Germany). Livers were removed and homogenized in PBS, and serial dilutions of the homogenates were plated on SBA to determine the amount of viable bacteria in the tissue, or used in ELISAs for cytokine determinations.

#### Cytokine determinations

Concentrations of murine IL-1β, IL-6, KC (the murine functional homolog to CXCL-1/IL-8), and TNFα in infected livers were determined by commercially available sandwich-type ELISAs, according to the manufacturer's instructions (R&D Systems, Wiesbaden-Nordenstadt, Germany).

#### Rabbit erythrocyte hemolysis assay

Rabbit blood agar (RBA) plates were prepared as follows: Fresh citrated rabbit blood (1 ml) was centrifuged at 5,000 × g for 5 min at room temperature, and sedimented cells were washed three times with 1 ml of 0.9% saline. After the last washing step, cells were suspended in 0.5 ml of 0.9% saline and 500 μl of the erythrocyte suspension was added to 20 ml of sterile Luria Bertani agar (Becton Dickinson) that was cooled to 50°C. Washed erythrocytes and Luria Bertani agar were gently mixed and poured into sterile petri dishes (Greiner Bio-One, Frickenhausen, Germany). If needed, RBA plates were supplemented with 250 μl of a sterile 40% glucose solution (final conc. 0.5%). Single colonies of *S. aureus* were streaked onto RBA plates and grown for 24 h at 37°C. After 24 h, images of the plates were obtained with a Leica D-Lux 3 in automatic mode.

#### Statistical analyses

The significance of changes between groups was assessed with the Mann–Whitney *U* test, using the GraphPad software package Prism 6.01. *P* < 0.05 were considered significant.

## Results

### Infectivity of *S. aureus* in normoglycemic mice is independent of CcpA in three different mouse models

**Figure 1:**
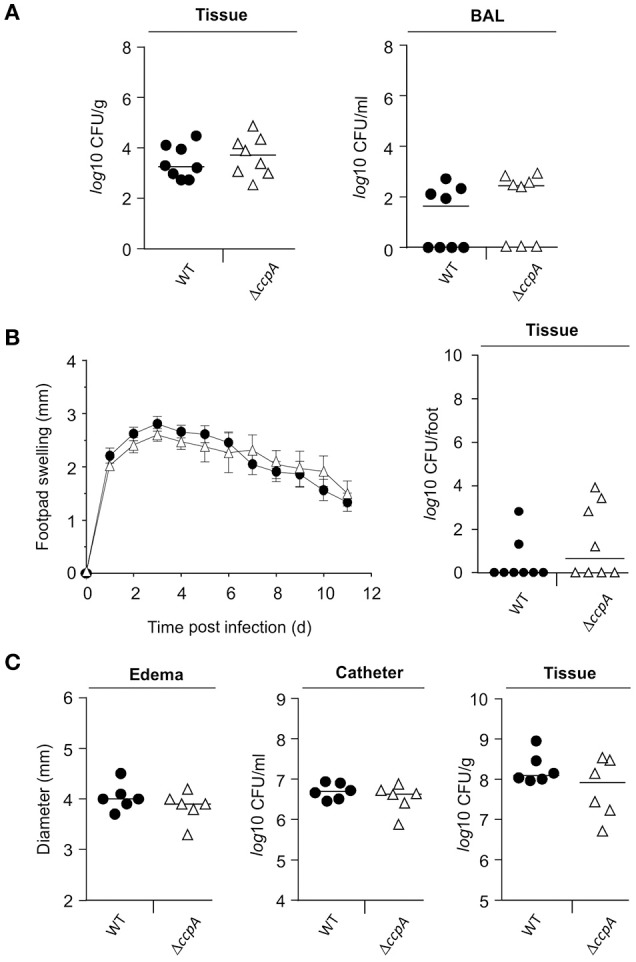
**Effect of a ***ccpA*** deletion on infectivity of ***S. aureus*** in normoglycemic C57BL/6N mice**. Normoglycemic female C57BL/6N mice were challenged with *S. aureus* strain Newman (black symbols) or strain MST14 (open symbols) in three infection models. Each symbol represents an individual animal, and horizontal bars indicate the median of all mice. **(A)** Bacterial loads in lung homogenates and in broncho-alveolar lavages (BALs) 24 h p.i. in a lung infection model (*n* = 8 per group). **(B)** Footpad swelling of *S. aureus*-infected mice in reference to the uninfected footpads (means ± SEM) and total bacterial loads in infected feet 11 days p.i. in a footpad infection model (*n* = 8 per group). **(C)** Size of edema and bacterial loads in tissue surrounding the catheters and in the catheters' lumen at day 11 p.i. in a catheter-related infection model (*n* = 6 per group).

Mice challenged with a *S. aureus ccpA* mutant displayed a reduced bacterial load in liver tissue in an abscess model (Li et al., 2010), suggesting that CcpA increases infectivity of *S. aureus*. To expand upon the *in vivo* function of CcpA in facilitating *S. aureus* infections, the effect of CcpA on infectivity was tested with an isogenic *S. aureus* strain pair Newman/MST14 (Newman Δ*ccpA*; Seidl et al., 2006) in three murine infection models: an acute pneumonia model (Hartmann et al., 2014), a subcutaneous footpad swelling model (Nippe et al., 2011), and a catheter-related infection model (Rupp et al., 1999). In contrast to the murine abscess model (Li et al., 2010), the infectivity of *S. aureus* wild-type strain Newman and the Δ*ccpA* mutant were equivalent in all three animal models when using normoglycemic C57BL/6N mice (Figure 1). Specifically, the bacterial loads in lung tissue and BAL were similar in the pneumonia model (Figure 1A); the swelling of footpads displayed comparable kinetics and maxima (Figure 1B); and the edema formation rates and bacterial loads in the catheter lumen and surrounding tissues were indistinguishable in the catheter infection model (Figure 1C). Taken together, these data demonstrate that under normoglycemic conditions infectivity of *S. aureus* is independent of CcpA.

### CcpA increases the bacterial load in liver tissue of *S. aureus* challenged mice

**Figure 2:**
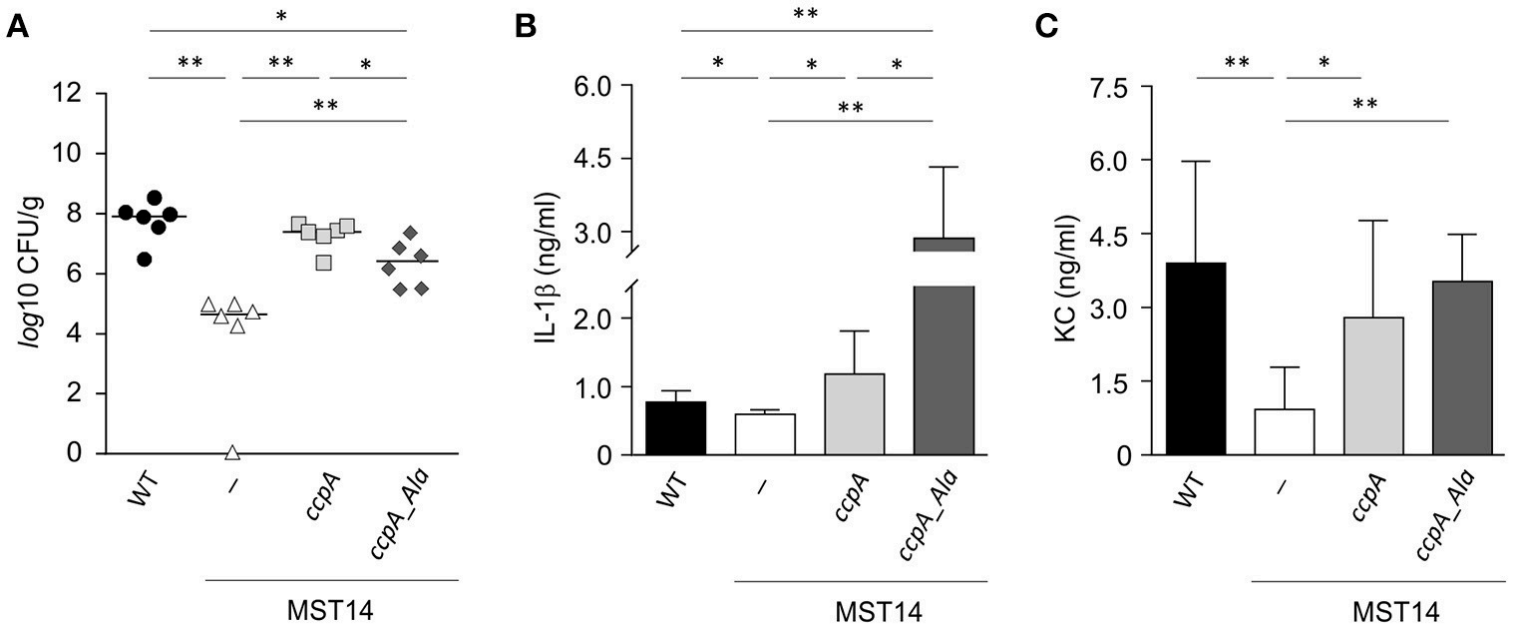
**Stk1 phosphorylation of CcpA alters ***S. aureus*** infectivity in a murine abscess model**. C57BL/6N mice (*n* = 6 per group) were challenged retro-orbitally with 1 × 10^7^ CFU of *S. aureus* strains Newman (black symbols), MST14 (white symbols), MST14 harboring plasmid pCN34_*ccpA* (light gray symbols), and MST14 harboring plasmid pCN34_*ccpA*_Ala (dark gray symbols), respectively, and mice were sacrificed 4 days p.i. **(A)** Bacterial loads determined by plating serial dilutions of homogenized tissue samples on sheep blood agar plates. **(B,C)** Cytokine levels of IL-1β **(B)** and KC [CXCL-1; **(C)**] in homogenized liver tissue of infected animals at 4 days p.i. are depicted. Data given in **(B)** and **(C)** represent the mean ± SD (*n* = 6). ^*^P < 0.05; ^**^P < 0.01 (Mann–Whitney U-test).

Strain Newman and the isogenic *ccpA* mutant caused equivalent infections in three mouse models, which differs from that reported by Li et al. (2010). To determine if this difference was due to the animal model or to differences in the type of *ccpA* mutation (i.e., deletion mutant vs. transposon mutant), the infectivity of strains Newman and the Δ*ccpA* mutant in the murine abscess model was assessed (Li et al., 2010). Consistent with the findings of Li et al. (2010), a significant decrease (about 3-log) in the bacterial loads in liver was observed in mice challenged with the Δ*ccpA* mutant strain MST14 as compared to mice infected with the wild-type strain Newman (Figure 2A). Mice challenged with a *trans*-complemented MST14 derivative harboring plasmid pCN34_*ccpA* displayed bacterial loads in liver comparable to wild-type infected animals (1.1 × 10^8^ ± 9.7 × 10^7^ CFU/g vs. 3.2 × 10^7^ ± 1.8 × 10^7^ CFU/g; *P* = 0.238). These data confirm the previous observations and demonstrate that decreased bacterial burden in liver of mice infected with *ccpA* mutants was due to the absence of CcpA. In addition, the data raise the question as to why CcpA is important in liver abscesses, but dispensable in other organs or anatomical sites?

### CcpA alters the cytokine response in liver tissue of *S. aureus* challenged mice

In systemic infections, *S. aureus* elicits a strong pro-inflammatory response by triggering the production and release of interleukins (IL)-1β (IL-1β), IL-6, IL-8, IL-18, and tumor necrosis factor alpha (TNFα) in immune and non-immune cells (Cui et al., 2000; Feezor et al., 2003; Hessle et al., 2005; Muller-Anstett et al., 2010). In *S. aureus ccpA* mutant challenged mice, the decreased bacterial loads in the liver raised the question of whether the lack of CcpA might also affect the innate immune response of the infected mice. To test this hypothesis, the concentrations of IL-1β, IL-6, TNFα, and the chemokine keratinocyte chemo-attractant (KC, syn. CXCL-1) were determined in mouse liver homogenates 4 days post-infection (Figures 2B,C and Figure S1). As expected, the concentrations of IL-1β (Figure 2B) and KC (Figure 2C) in the liver homogenates of MST14 challenged mice were significantly decreased relative to wild-type infected mice. Similarly, the cytokines IL-6 and TNFα were decreased in MST14 challenged mice; however, these effects were not statistically significant (Figure S1). Upon challenge of mice with the *ccpA* complemented MST14 derivative (MST14 pCN34_*ccpA*), the cytokine concentrations in liver tissue were restored to levels similar to mice infected with the wild-type strain (Figure 2B). Taken together, these data suggest that *ccpA*-positive *S. aureus* provoke an increased pro-inflammatory response in infected liver tissue.

### The phosphoablative *ccpA* variant Ccpa_T18A/T33A affects the pathophysiology of *S. aureus* in the murine abscess model

Recently, it was determined that the DNA binding activity of CcpA in *S. aureus* is controlled by the serine/threonine kinase Stk1 (Leiba et al., 2012). To test whether the Stk1-dependent phosphorylation of CcpA would affect the infectivity of *S. aureus*, C57BL/6N mice were challenged with a MST14 derivative *trans*-complemented with a *ccpA* allele having threonine to alanine mutations at positions 18 and 33 (MST14 pCN34_*ccpA*_Ala) and the bacterial load and cytokine profiles were determined. This CcpA_T18A/T33A variant can no longer be phosphorylated by Stk1, but retains the regulatory activity toward its target genes (Leiba et al., 2012). Interestingly, while the bacterial loads in livers of mice infected with strain MST14 complemented with the wild-type *ccpA* allele were similar to those of mice challenged with the wild-type strain, the infection progress differed in mice infected with MST14 pCN34_*ccpA*_Ala (Figure 2A). Specifically, mice challenged with MST14 pCN34_*ccpA*_Ala significantly increased the bacterial load in livers by a factor of ~1.8-log relative to the MST14 infected mice, but remained ~1.2- and ~0.7-log below the levels of the wild-type and MST14 pCN34_*ccpA* strains, respectively (Figure 2A). Similarly, infecting mice with strain MST14 pCN34_*ccpA*_Ala resulted in a significant increase in the IL-1β levels in liver relative to that observed in mice challenged with the wild-type or MST14 pCN34_*ccpA* strains (Figure 2B). In contrast, KC levels were indistinguishable between these three groups (Figure 2C). Taken together, under *in vivo* conditions the Stk1-dependent phosphorylation of CcpA dramatically alters the infection process.

### Ccpa alters the infectivity of *S. aureus* in a hyperglycemic environment

**Figure 3:**
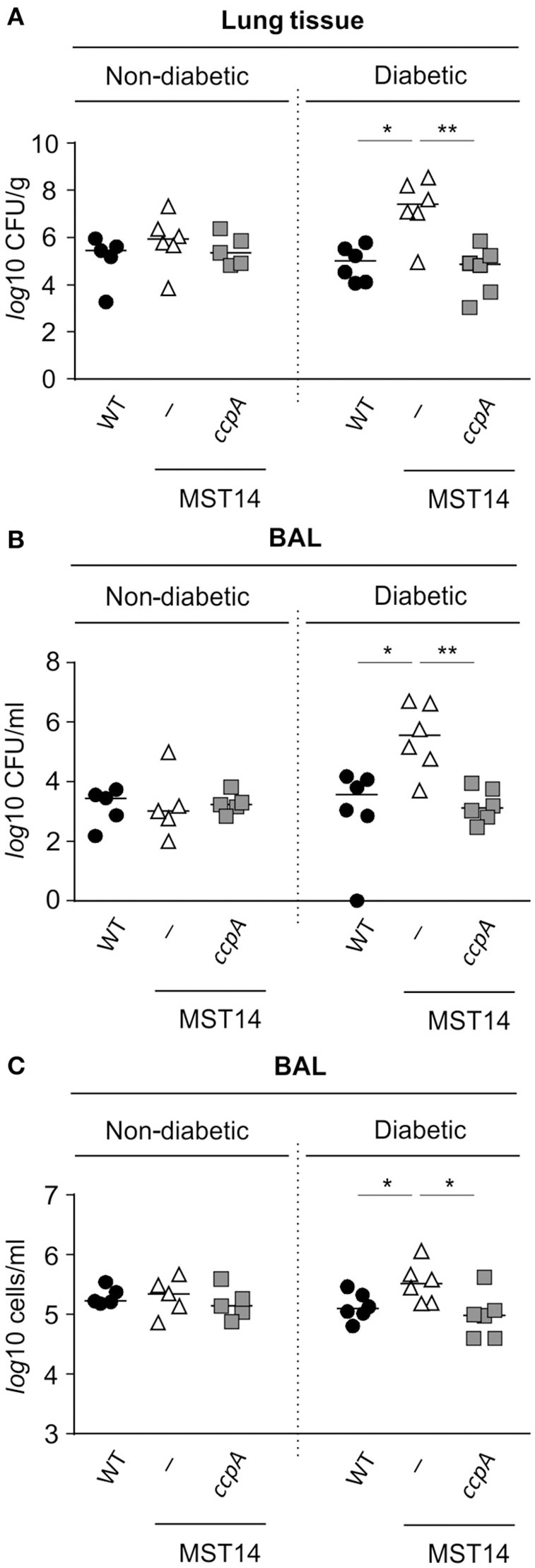
**Effect of a ***ccpA*** deletion on infectivity of ***S. aureus*** in NOD mice in an acute pneumonia model**. Age-matched diabetic and non-diabetic NOD mice were infected intranasally with 5 × 10^7^ CFUs of *S. aureus* strain Newman (black symbols), its Δ*ccpA* derivative MST14 (white symbols), and MST14 *trans*-complemented with plasmid pCN35_*ccpA* (gray symbols), respectively (*n* = 5–6 per group). Mice were euthanized 24 h p.i., and bacterial loads in homogenized lung tissue **(A)** and BALs **(B)** determined. In addition, the total eukaryotic immune cells in BALs were investigated **(C)**. Each symbol represents an individual animal, and horizontal bars indicate the median of all observations. ^*^P < 0.05; ^**^P < 0.01 (Mann–Whitney U-test).

As mentioned, mice challenged with a *ccpA* mutant had decreased bacterial numbers in the liver (Figure 2A), but not in kidneys (Li et al., 2010). This raised the question as to why the effect of *ccpA* inactivation was specific to the liver. Notably, glucose concentrations in the liver are elevated in comparison to those found in blood and intestinal fluids of the same animals (Appelboom et al., 1959; Wals and Katz, 1993). These observations suggest that CcpA might contribute to the infectivity of *S. aureus* in hyperglycemic individuals. To address this hypothesis, the infectivity of strains Newman, MST14, and MST14 pCN34_*ccpA* was assessed in an acute pneumonia model using female NOD mice that spontaneously develop a type 1 diabetes between 15 and 30 weeks of age (Leiter, 2001). Consistent with the pneumonia model using C57BL/6N mice (Figure 1A), challenging normoglycemic NOD mice (blood glucose level ≤ 10 mM) with any of the three strains resulted in equivalent CFU counts in lung tissue homogenates (Figure 3A) and BAL (Figure 3B) 24 h post-infection. Similarly, the total eukaryotic immune cell counts in BAL were comparable between all groups (Figure 3C), indicating an analogical course of infection caused by the three *S. aureus* strains. In contrast, infection of age-matched diabetic NOD mice (blood glucose level ≥20 mM) with the Δ*ccpA* mutant MST14 significantly increased the bacterial loads relative to the wild-type strain in lung tissue (~2.7-log; Figure 3A) and BALs (~2.5-log; Figure 3B). This was accompanied by a significant increase in total immune cells in BALs of the MST14 challenged mice (Figure 3C), indicating an increased severity of infection. Complementation of the Δ*ccpA* mutant with plasmid pCN34_*ccpA* restored all virulence traits to wild-type levels, confirming that the alterations observed with MST14 were caused by inactivation of *ccpA*.

**Figure 4:**
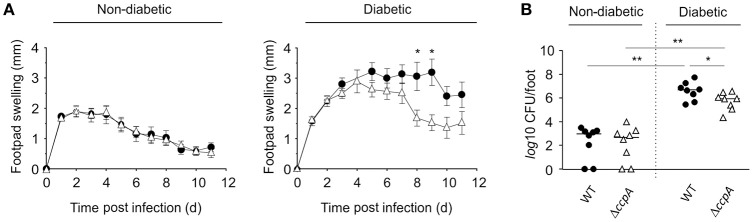
**Effect of a ***ccpA*** deletion on infectivity of ***S. aureus*** in NOD mice in a footpad swelling model**. Cells of *S. aureus* strain Newman (black symbols) and its Δ*ccpA* derivative MST14 (white symbols) were injected subcutaneously into the footpads of age-matched diabetic and non-diabetic NOD mice (*n* = 8 per group), respectively, and swelling of the footpads were measured on a daily basis in reference to the uninfected footpads. **(A)** Time course of footpad swelling of *S. aureus*-infected mice. Data shown represent the means ± SEM. **(B)** Bacterial loads of *S. aureus* strain Newman (black symbols) and MST14 (white symbols) in infected feet 11 days p.i. Each symbol represents an individual animal, and horizontal bars indicate the median of all observations. ^*^P < 0.05; ^**^P < 0.01 (Mann–Whitney U-test).

To determine if CcpA was important for extrapulmonary *S. aureus* infections in diabetic NOD mice, strain Newman and MST14 were evaluated in the footpad swelling model and catheter related infection models. The pCN34_*ccpA* harboring MST14 derivative was not included, as maintenance of the pT181-derived plasmid within the *trans*-complemented derivative was not assured over the entire time course of these *in vivo* models (Krute et al., 2016). Similar to our findings with the pneumonia model, the wild-type and Δ*ccpA* mutant infected normoglycemic mice displayed comparable footpad swelling kinetics (Figure 4A), and both strains persisted in the infected foot tissue for a comparable number of 11 days p.i. (Figure 4B). In contrast, when hyperglycemic NOD mice were infected with strains Newman and MST14, clear differences in the footpad swelling kinetics were observed (Figure 4A). The footpads of mice infected with the wild-type strain Newman showed enhanced swelling rates 5 days p.i. that were significantly increased at days 8 and 9 when compared to the footpad swelling rates of the strain MST14 challenged mice. Consistent with increased footpad swelling, significantly greater numbers of bacteria (~7.1-fold) were recovered from the tissue homogenates of infected feet of mice challenged with wild-type *S. aureus* in comparison to mice infected with the Δ*ccpA* mutant (Figure 4B).

**Figure 5:**
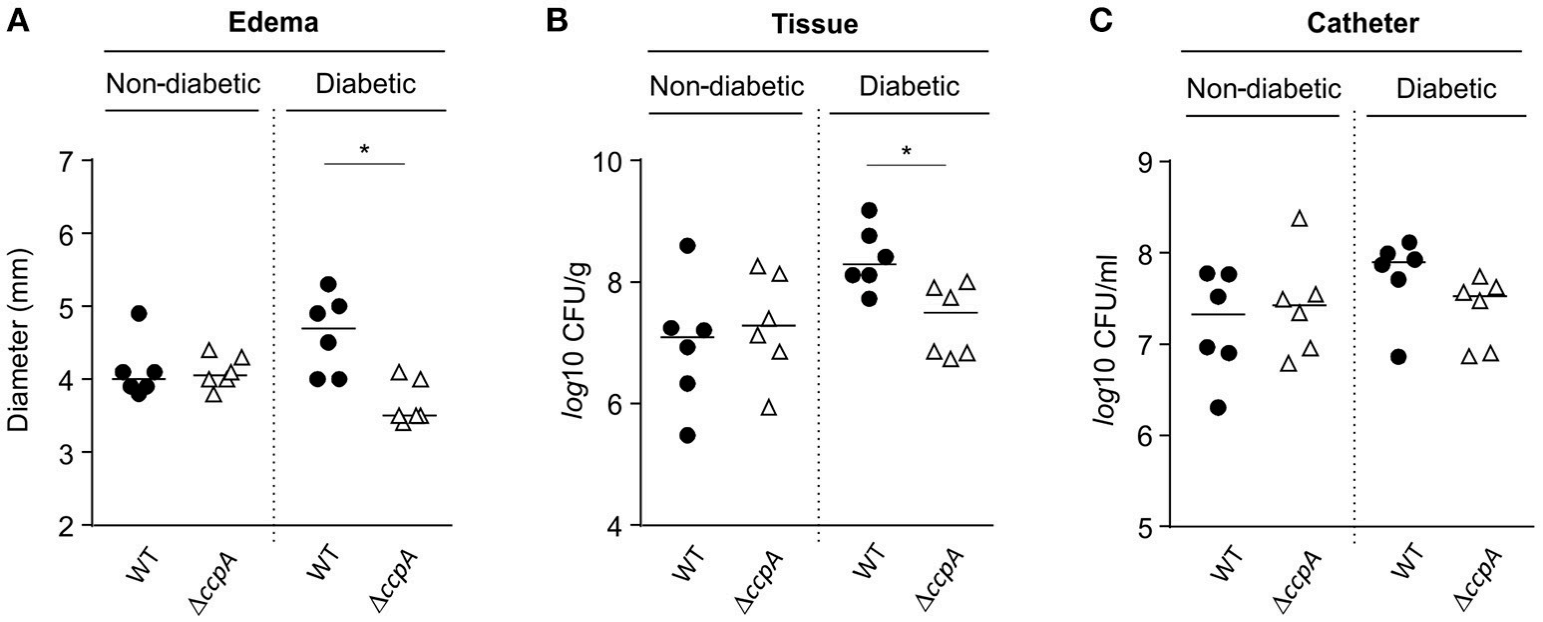
**Effect of CcpA on infectivity of ***S. aureus*** in NOD mice in a catheter-related infection model**. Sterile catheter segments were inserted subcutaneously into diabetic and non-diabetic NOD mice (*n* = 6 per group) and subsequently infected with cells of *S. aureus* strain Newman (black symbols) and MST14 (white symbols). On day 11 after infection, edema end points at the insertion site were determined, and the catheters and the surrounding tissues removed and separated. Bacteria adherent to the catheters were detached by vortexing in saline, and tissue samples were homogenized in saline. Edema end points at day 11 p.i. **(A)**, and bacterial loads in tissues surrounding the catheters **(B)** and in catheter lumen 11 days p.i. **(C)** are depicted. Each symbol represents an individual animal, and horizontal bars indicate the median of all observations. ^*^P < 0.05 (Mann–Whitney U-test).

Similar to the footpad swelling model, infection symptoms in mice infected with strain Newman and MST14 were equivalent in normoglycemic NOD mice using a catheter related infection model (Rupp et al., 1999; Figure 5). In contrast, infection of diabetic NOD mice with the wild-type strain Newman significantly increased edema (Figure 5A) and enhanced bacterial loads in the tissue surrounding the catheter (~1-log; Figure 5B) compared to mice infected with the Δ*ccpA* mutant strain MST14. In total, these data suggest that CcpA is critically important for the pathogenesis of *S. aureus* in hyperglycemic individuals.

### CcpA promotes hemolysis in a glucose-rich environment

**Figure 6:**
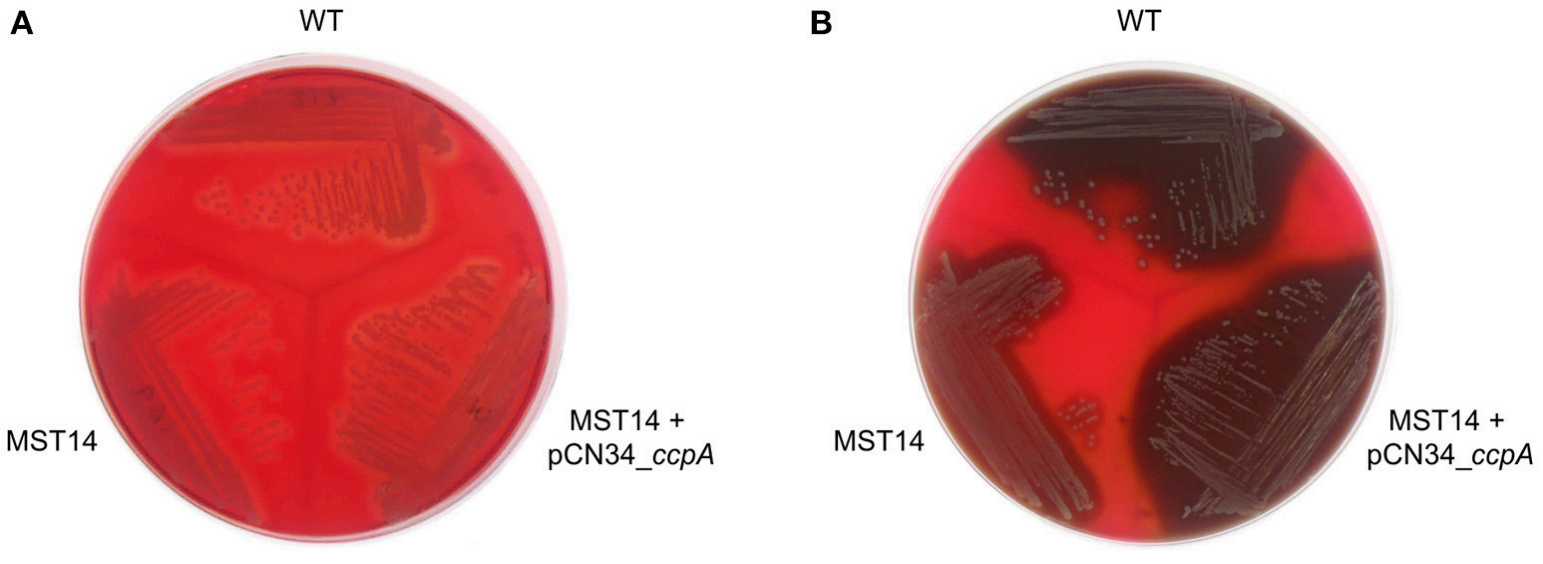
**Effect of CcpA on the hemolytic activity of ***S. aureus*****. Single colonies of *S. aureus* strains Newman (WT), MST14 (Newman Δ*ccpA*), and MST14 complemented with plasmid pCN34_*ccpA*, respectively, were streaked onto rabbit blood agar plates in the absence **(A)** or presence of 0.5% glucose **(B)**, and grown for 24 h at 37°C. The photographs are representative of three independent experiments. The image depicted in A was recorded in back-light mode to visualize the hemolytic areas.

CcpA increases the transcription of the α-hemolysin encoding gene *hla* in a glucose-responsive manner (Seidl et al., 2006; Leiba et al., 2012), and this toxin is a major virulence factor of *S. aureus* (reviewed in Vandenesch et al., 2012). To determine whether CcpA-mediated changes in *hla* transcription translate into a greater α-hemolysin accumulation, the capacities of *S. aureus* strain Newman and its *ccpA* derivatives to lyse rabbit erythrocytes were determined. Rabbit erythrocytes are particularly sensitive to the pore-forming toxin α-hemolysin (Cooper et al., 1966). When *S. aureus* strains Newman, MST14, and MST14 harboring pCN34_*ccpA* were streaked onto RBA plates lacking glucose, all three strains produced small and comparable hemolytic zones surrounding the growing colonies (Figure 6A). In contrast, when the three strains were streaked onto RBA plates supplemented with 0.5% glucose (~28 mM), strains Newman and MST14 harboring pCN34_*ccpA* produced larger hemolytic areas than strain MST14 (Figure 6B), suggesting that CcpA augments α-hemolysin accumulation in a glucose-rich environment.

## Discussion

Pathogenic bacteria commonly link virulence determinant synthesis with central metabolic pathways (Eisenreich et al., 2010; Rohmer et al., 2011; Richardson et al., 2015). In *S. aureus*, this linkage is mediated via a number of catabolite-responsive regulatory proteins, such as CcpA (Seidl et al., 2006), CcpE (Hartmann et al., 2014), CodY (Montgomery et al., 2012), and the RpiR homologs RpiRb and RpiRc (Zhu et al., 2011). For most of these regulators (i.e., CcpE, CodY, and RpiRc), an attenuating effect on infectivity of *S. aureus* is observed in murine infection models (Montgomery et al., 2012; Ding et al., 2014; Hartmann et al., 2014; Balasubramanian et al., 2016; Gaupp et al., 2016). Interestingly, only the glucose-responsive regulator CcpA positively contributes to the infectivity of *S. aureus* in mice (Li et al., 2010). *In vitro* findings demonstrate that 4 mM of glucose is sufficient to fully activate CcpA (Seidl et al., 2008a), suggesting it is active in normoglycemic mice with fasting blood glucose levels of 7–9 mM (Berglund et al., 2008). However, the *in vivo* observation of Li et al. (2010), indicating a negative effect of *ccpA* inactivation on bacterial loads in liver but not kidneys, suggest that CcpA might require elevated glucose levels to modulate the infectivity of *S. aureus*. This suggestion is consistent with our observations regarding the contribution of CcpA to infectivity of *S. aureus* under normo- and hyperglycemic conditions; specifically, CcpA's influence is only realized under elevated glucose conditions.

While the influence of *S. aureus* CcpA on infectivity is predominantly seen in diabetic mice, the effects of CcpA on pathogenesis differ between disease niches. Specifically, CcpA functions as an attenuator of *S. aureus* virulence in the pneumonia model (Figure 3), while in the footpad swelling model (Figure 4) and the catheter related infection model (Figure 5) inactivation of *ccpA* significantly decreased infection symptoms. While the mechanism(s) by which CcpA mediates differential virulence responses in different niches remains speculative, *in vitro* data provide clues to the nature of this mechanism(s).

CcpA enhances the hemolytic potential of *S. aureus* in a glucose-rich environment (Figure 6), and promotes transcription of the α-hemolysin gene *hla* during post-exponential growth *in vitro* (Seidl et al., 2006; Leiba et al., 2012). This secreted pore-forming exotoxin is crucial in *S. aureus* skin and soft tissue infections (SSTI; reviewed in Kobayashi et al., 2015). In particular, α-hemolysin destabilizes the dermis by inducing tissue barrier disruption and host cell cytolysis, and increasing the level of the pro-inflammatory cytokine IL-1β in infected tissue, which induces a neutrophil influx to the site of infection (reviewed in Berube and Bubeck Wardenburg, 2013). Together, reduced α-hemolysin production by the Δ*ccpA* mutant, strain MST14, likely contributes to the attenuation of infection symptoms in the footpad swelling model and the catheter related infection model in hyperglycemic NOD mice (Figures 4, 5). Decreased α-hemolysin synthesis would also contribute to decreased CFU and Il-1β and KC concentrations observed in livers of strain MST14 infected C57BL/6 mice (Figure 2). This would also explain the augmented Il-1β concentrations found in liver homogenates of mice infected with MST14 pCN34 *ccpA*_Ala (Figure 2B). Namely, complementation of strain MST14 with the mutated *ccpA*_Ala plasmid prevents the Stk1 facilitated phosphorylation of CcpA, which blocks CcpA binding to the *hla* promoter (Leiba et al., 2012), thereby increasing α-hemolysin synthesis and subsequently the levels of IL-1β in liver (Figure 2B). Taken together, it is likely that the main effect of CcpA in these animal models is exerted through changes in α-hemolysin synthesis.

In *S. aureus*, α-hemolysin and protein A (SpA) are inversely regulated (Goerke et al., 2000; Oogai et al., 2011; Gao et al., 2016). In other words, when α-hemolysin synthesis is elevated, SpA synthesis is decreased. This is significant because SpA is important for the infectivity of *S. aureus* in lung infection models (Gomez et al., 2004; Bubeck Wardenburg et al., 2007). SpA activates and controls the pro-inflammatory response by interacting with the TNF receptor 1 and the epidermal growth factor receptor on airway epithelial cells, which contributes to the pathogenesis of staphylococcal pneumonia (reviewed in Parker and Prince, 2012). Importantly, *spa* transcription is strongly repressed by CcpA in response to glucose availability (Seidl et al., 2006). Based on these observations, the increased bacterial loads in the lungs and BALs of diabetic mice challenged with MST14 are likely due to enhanced synthesis of SpA by the Δ*ccpA* mutant (Figure 3). More importantly, these data suggest the inverse relationship between α-hemolysin and SpA contributes to disease severity within different niches.

## Ethics statement

This study was carried out in accordance with the regulations of German and Swiss veterinary law, respectively. All animal studies were performed with the approval of the animal welfare committees Landesamt für Verbraucherschutz (Saarbrucken, Germany), Landesamt für Natur Umwelt und Verbraucherschutz (Recklinghausen, Germany), and cantonal veterinary office of Basel-Stadt (Switzerland), respectively.

## Author contributions

MB, CB, CS, VM, QD, FL, RB, MH, GS, and TT designed the research. MB, NN, NJN, and MV analyzed data. MB, BW, NN, NJN, and MV were responsible for the experimental work. MB, GS, and RG wrote the manuscript. All authors approved the manuscript.

## Funding

This study was supported by the German Research Foundation grants Bi 1350/1-1 and Bi 1350/1-2 to MB and MH, and NN and CS were supported by BMBF grant SkinStaph (No. 01Kl07100).

### Conflict of interest statement

The authors declare that the research was conducted in the absence of any commercial or financial relationships that could be construed as a potential conflict of interest.
