# Supplementary material for: CcpA Affects Infectivity of Staphylococcus aureus in a Hyperglycemic Environment
Source: Front Cell Infect Microbiol. 2017 May 9;7:172. doi: 10.3389/fcimb.2017.00172 (PMC5422431; doi:10.3389/fcimb.2017.00172)
Supplement: Supplementary file 1 [file Image1.PDF]

Fig: S1

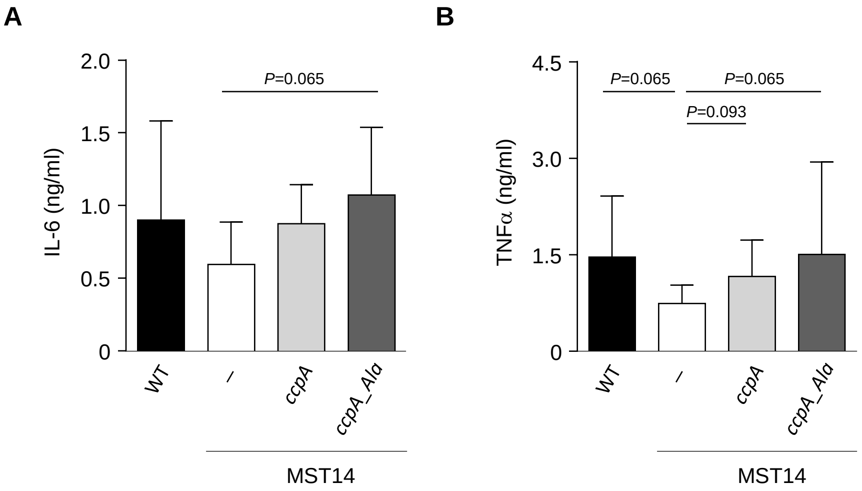

Figure S1: Cytokine level in liver tissue of *S. aureus* infected C57BL/6N mice four days post infection. C57BL/6N mice were challenged retro-orbitally with 1 x 10^7 CFU of *S. aureus* strains Newman (black symbols), MST14 (white symbols), MST14 harboring plasmid pCN34_*ccpA* (light grey symbols), and MST14 harboring plasmid pCN34_*ccpA*_Ala (dark grey symbols), respectively. Mice were sacrificed four days post infection, and cytokine levels in liver homogenates were determined by ELISA. **A)** Interleukin 6 (IL-6) levels in liver tissue of infected animals four days post infection. **B)** Tumor necrosis factor α (TNFα) levels in liver tissue of infected animals four days post infection. Data represent the mean ± SD (n=6 mice). *P* values <0.1 are indicated (Mann–Whitney U test).
